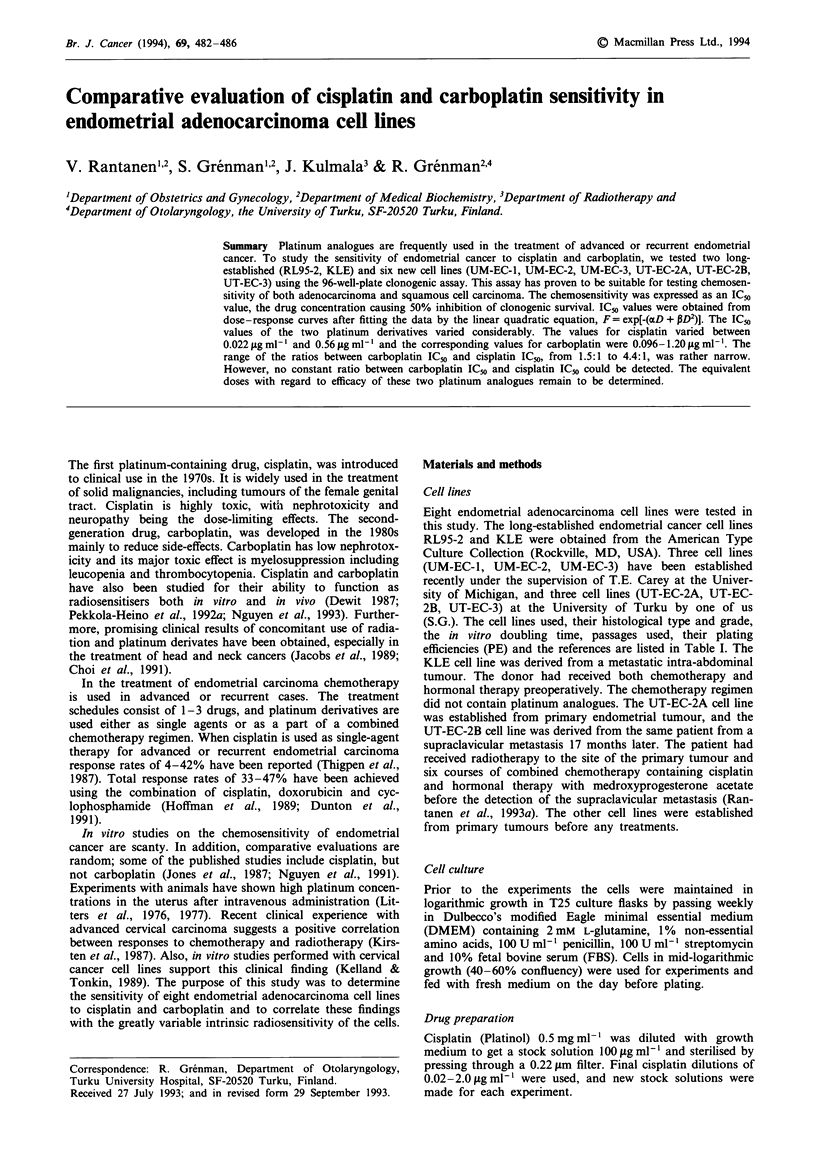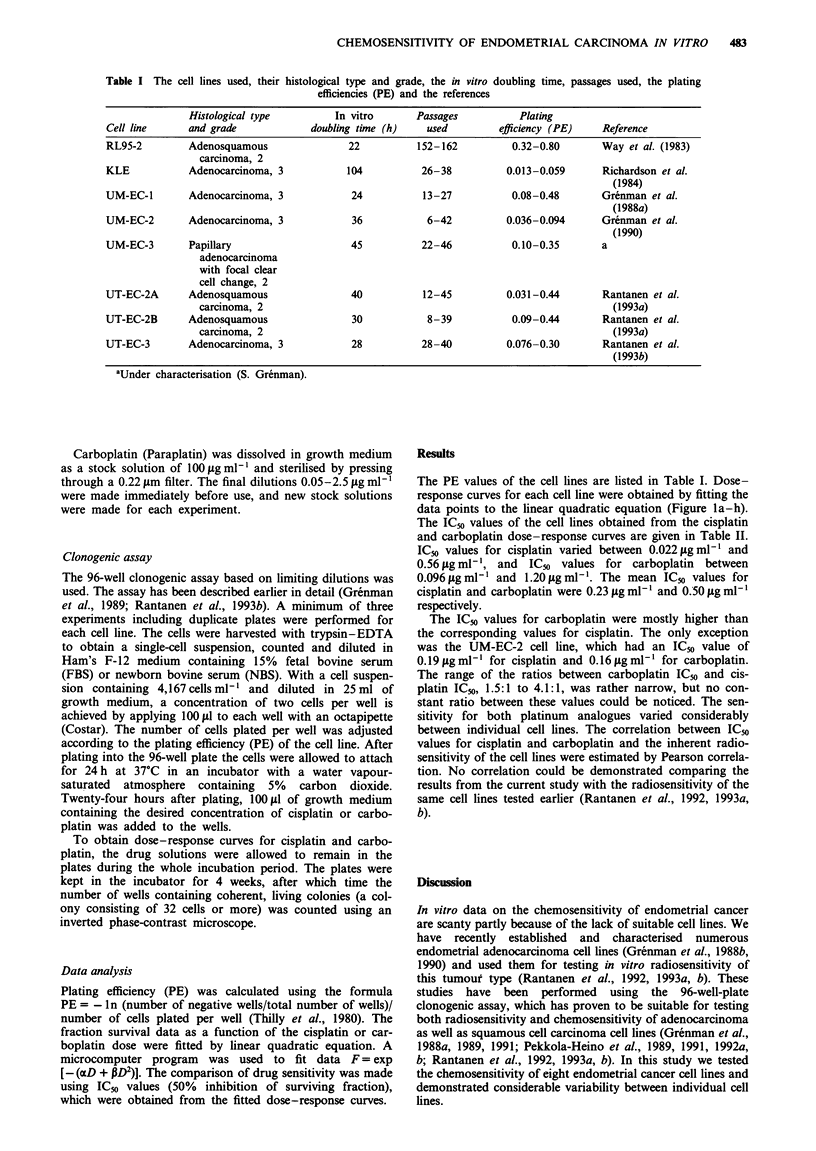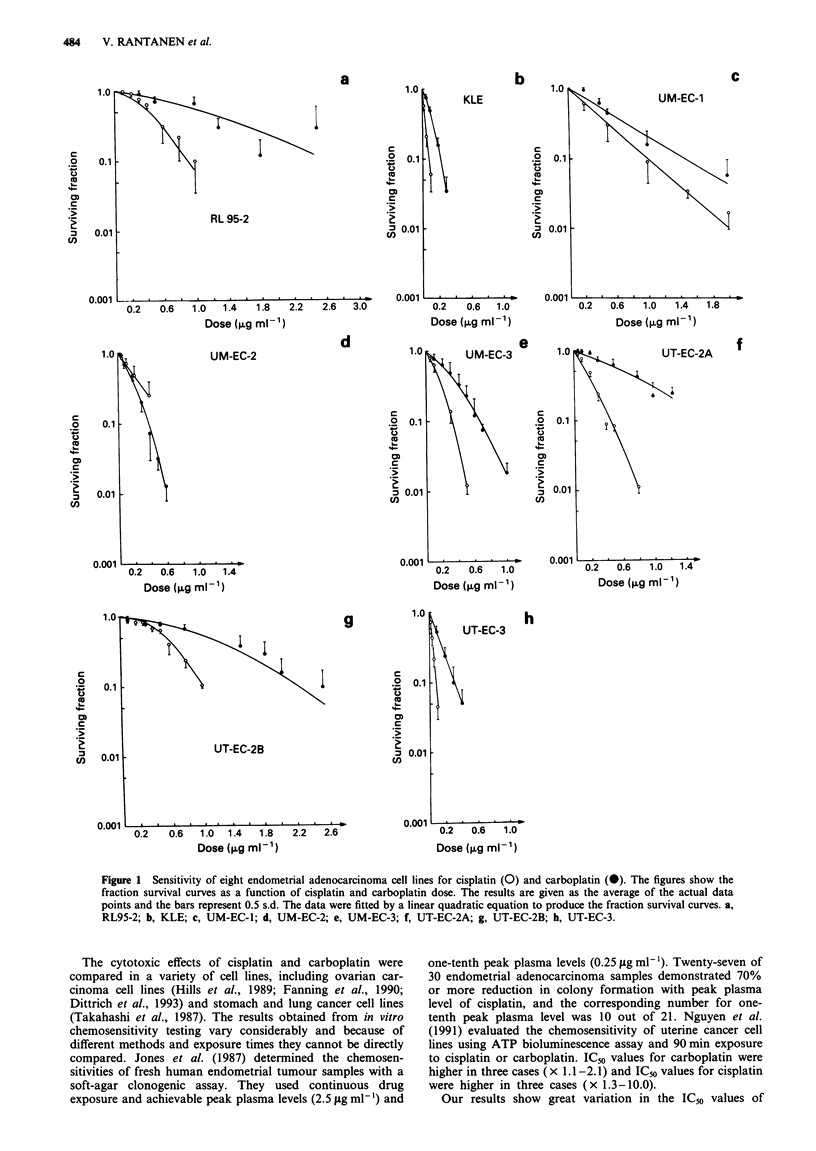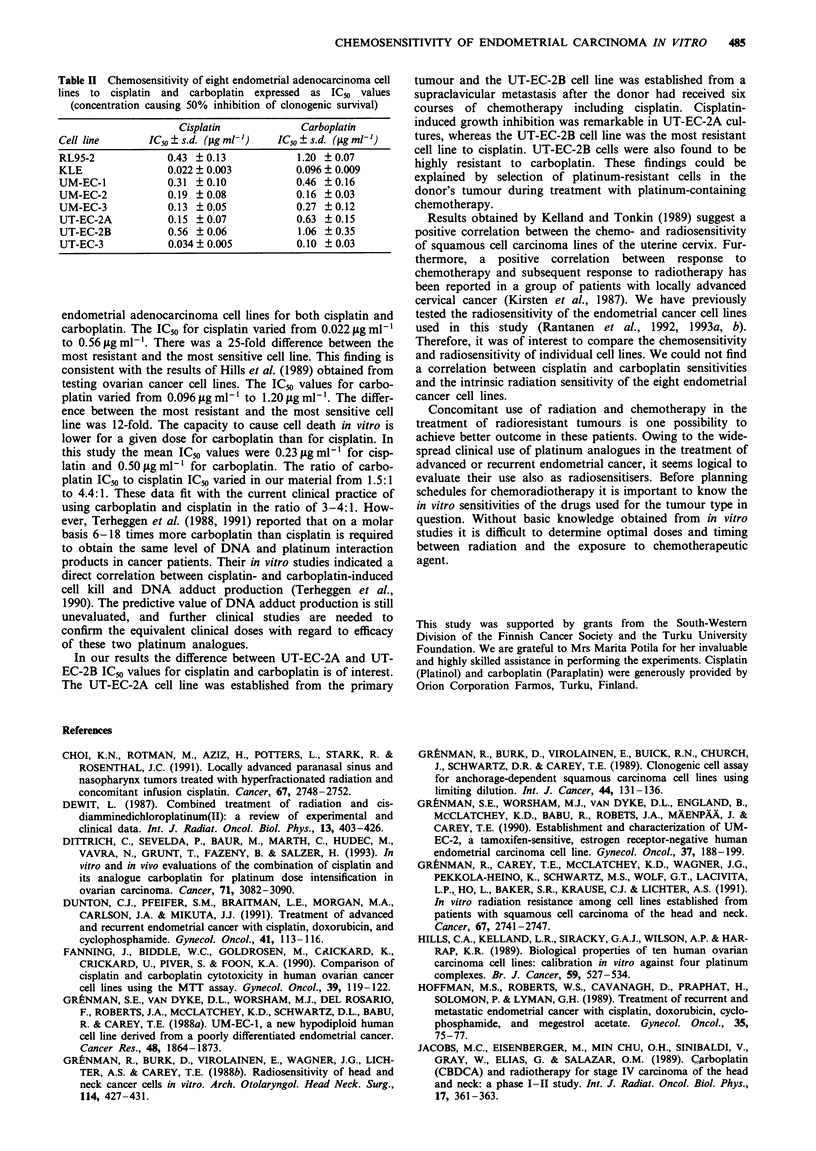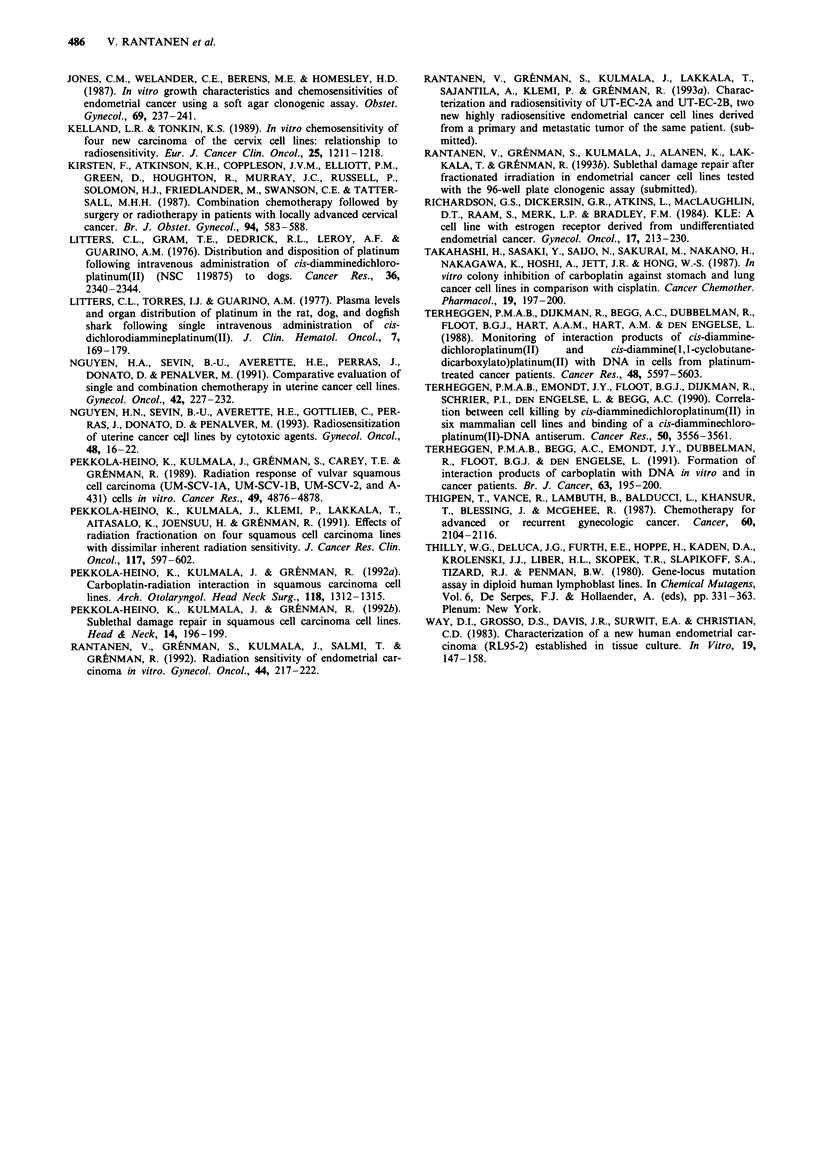# Comparative evaluation of cisplatin and carboplatin sensitivity in endometrial adenocarcinoma cell lines.

**DOI:** 10.1038/bjc.1994.87

**Published:** 1994-03

**Authors:** V. Rantanen, S. Grénman, J. Kulmala, R. Grénman

**Affiliations:** Department of Obstetrics and Gynecology, University of Turku, Finland.

## Abstract

Platinum analogues are frequently used in the treatment of advanced or recurrent endometrial cancer. To study the sensitivity of endometrial cancer to cisplatin and carboplatin, we tested two long-established (RL95-2, KLE) and six new cell lines (UM-EC-1, UM-EC-2, UM-EC-3, UT-EC-2A, UT-EC-2B, UT-EC-3) using the 96-well-plate clonogenic assay. This assay has proven to be suitable for testing chemosensitivity of both adenocarcinoma and squamous cell carcinoma. The chemosensitivity was expressed as an IC50 value, the drug concentration causing 50% inhibition of clonogenic survival. IC50 values were obtained from dose-response curves after fitting the data by the linear quadratic equation, F = exp[-(alpha D + beta D2)]. The IC50 values of the two platinum derivatives varied considerably. The values for cisplatin varied between 0.022 microgram ml-1 and 0.56 microgram ml-1 and the corresponding values for carboplatin were 0.096-1.20 microgram ml-1. The range of the ratios between carboplatin IC50 and cisplatin IC50, from 1.5:1 to 4.4:1, was rather narrow. However, no constant ratio between carboplatin IC50 and cisplatin IC50 could be detected. The equivalent doses with regard to efficacy of these two platinum analogues remain to be determined.


Br. J. Cancer (1994), 69, 482-486 © Macmillan Press Ltd., 1994

### Comparative evaluation of cisplatin and carboplatin sensitivity in endometrial adenocarcinoma cell lines

V. Rantanen[1,2], S. Grénman[1,2], J. Kulmala[3] & R. Grénman[2,4]

[1]Department of Obstetrics and Gynecology, [2]Department of Medical Biochemistry, [3]Department of Radiotherapy and [4]Department of Otolaryngology, the University of Turku, SF-20520 Turku, Finland.

**Summary** Platinum analogues are frequently used in the treatment of advanced or recurrent endometrial cancer. To study the sensitivity of endometrial cancer to cisplatin and carboplatin, we tested two long-established (RL95-2, KLE) and six new cell lines (UM-EC-1, UM-EC-2, UM-EC-3, UT-EC-2A, UT-EC-2B, UT-EC-3) using the 96-well-plate clonogenic assay. This assay has proven to be suitable for testing chemosensitivity of both adenocarcinoma and squamous cell carcinoma. The chemosensitivity was expressed as an $IC_{50}$ value, the drug concentration causing 50% inhibition of clonogenic survival. $IC_{50}$ values were obtained from dose-response curves after fitting the data by the linear quadratic equation, $F = \exp[-(\alpha D + \beta D^2)]$. The $IC_{50}$ values of the two platinum derivatives varied considerably. The values for cisplatin varied between 0.022 $\mu g\ ml^{-1}$ and 0.56 $\mu g\ ml^{-1}$ and the corresponding values for carboplatin were 0.096-1.20 $\mu g\ ml^{-1}$. The range of the ratios between carboplatin $IC_{50}$ and cisplatin $IC_{50}$, from 1.5:1 to 4.4:1, was rather narrow. However, no constant ratio between carboplatin $IC_{50}$ and cisplatin $IC_{50}$ could be detected. The equivalent doses with regard to efficacy of these two platinum analogues remain to be determined.

The first platinum-containing drug, cisplatin, was introduced to clinical use in the 1970s. It is widely used in the treatment of solid malignancies, including tumours of the female genital tract. Cisplatin is highly toxic, with nephrotoxicity and neuropathy being the dose-limiting effects. The second-generation drug, carboplatin, was developed in the 1980s mainly to reduce side-effects. Carboplatin has low nephrotoxicity and its major toxic effect is myelosuppression including leucopenia and thrombocytopenia. Cisplatin and carboplatin have also been studied for their ability to function as radiosensitisers both *in vitro* and *in vivo* (Dewit 1987; Pekkola-Heino *et al*., 1992*a*; Nguyen *et al*., 1993). Furthermore, promising clinical results of concomitant use of radiation and platinum derivates have been obtained, especially in the treatment of head and neck cancers (Jacobs *et al*., 1989; Choi *et al*., 1991).

In the treatment of endometrial carcinoma chemotherapy is used in advanced or recurrent cases. The treatment schedules consist of 1-3 drugs, and platinum derivatives are used either as single agents or as a part of a combined chemotherapy regimen. When cisplatin is used as single-agent therapy for advanced or recurrent endometrial carcinoma response rates of 4-42% have been reported (Thigpen *et al*., 1987). Total response rates of 33-47% have been achieved using the combination of cisplatin, doxorubicin and cyclophosphamide (Hoffman *et al*., 1989; Dunton *et al*., 1991).

*In vitro* studies on the chemosensitivity of endometrial cancer are scanty. In addition, comparative evaluations are random; some of the published studies include cisplatin, but not carboplatin (Jones *et al*., 1987; Nguyen *et al*., 1991). Experiments with animals have shown high platinum concentrations in the uterus after intravenous administration (Litters *et al*., 1976, 1977). Recent clinical experience with advanced cervical carcinoma suggests a positive correlation between responses to chemotherapy and radiotherapy (Kirsten *et al*., 1987). Also, *in vitro* studies performed with cervical cancer cell lines support this clinical finding (Kelland & Tonkin, 1989). The purpose of this study was to determine the sensitivity of eight endometrial adenocarcinoma cell lines to cisplatin and carboplatin and to correlate these findings with the greatly variable intrinsic radiosensitivity of the cells.

Correspondence: R. Grénman, Department of Otolaryngology, Turku University Hospital, SF-20520 Turku, Finland.
Received 27 July 1993; and in revised form 29 September 1993.

### Materials and methods

### Cell lines

Eight endometrial adenocarcinoma cell lines were tested in this study. The long-established endometrial cancer cell lines RL95-2 and KLE were obtained from the American Type Culture Collection (Rockville, MD, USA). Three cell lines (UM-EC-1, UM-EC-2, UM-EC-3) have been established recently under the supervision of T.E. Carey at the University of Michigan, and three cell lines (UT-EC-2A, UT-EC-2B, UT-EC-3) at the University of Turku by one of us (S.G.). The cell lines used, their histological type and grade, the *in vitro* doubling time, passages used, their plating efficiencies (PE) and the references are listed in Table I. The KLE cell line was derived from a metastatic intra-abdominal tumour. The donor had received both chemotherapy and hormonal therapy preoperatively. The chemotherapy regimen did not contain platinum analogues. The UT-EC-2A cell line was established from primary endometrial tumour, and the UT-EC-2B cell line was derived from the same patient from a supraclavicular metastasis 17 months later. The patient had received radiotherapy to the site of the primary tumour and six courses of combined chemotherapy containing cisplatin and hormonal therapy with medroxyprogesterone acetate before the detection of the supraclavicular metastasis (Rantanen *et al*., 1993*a*). The other cell lines were established from primary tumours before any treatments.

### Cell culture

Prior to the experiments the cells were maintained in logarithmic growth in T25 culture flasks by passing weekly in Dulbecco's modified Eagle minimal essential medium (DMEM) containing 2 mM L-glutamine, 1% non-essential amino acids, 100 U $ml^{-1}$ penicillin, 100 U $ml^{-1}$ streptomycin and 10% fetal bovine serum (FBS). Cells in mid-logarithmic growth (40-60% confluency) were used for experiments and fed with fresh medium on the day before plating.

### Drug preparation

Cisplatin (Platinol) 0.5 mg $ml^{-1}$ was diluted with growth medium to get a stock solution 100 $\mu g\ ml^{-1}$ and sterilised by pressing through a 0.22 $\mu m$ filter. Final cisplatin dilutions of 0.02-2.0 $\mu g\ ml^{-1}$ were used, and new stock solutions were made for each experiment.

**Table I** The cell lines used, their histological type and grade, the *in vitro* doubling time, passages used, the plating efficiencies (PE) and the references

| *Cell line* | *Histological type and grade* | *In vitro doubling time (h)* | *Passages used* | *Plating efficiency (PE)* | *Reference* |
|---|---|---|---|---|---|
| RL95-2 | Adenosquamous carcinoma, 2 | 22 | 152–162 | 0.32–0.80 | Way *et al.* (1983) |
| KLE | Adenocarcinoma, 3 | 104 | 26–38 | 0.013–0.059 | Richardson *et al.* (1984) |
| UM-EC-1 | Adenocarcinoma, 3 | 24 | 13–27 | 0.08–0.48 | Grénman *et al.* (1988*a*) |
| UM-EC-2 | Adenocarcinoma, 3 | 36 | 6–42 | 0.036–0.094 | Grénman *et al.* (1990) |
| UM-EC-3 | Papillary adenocarcinoma with focal clear cell change, 2 | 45 | 22–46 | 0.10–0.35 | a |
| UT-EC-2A | Adenosquamous carcinoma, 2 | 40 | 12–45 | 0.031–0.44 | Rantanen *et al.* (1993*a*) |
| UT-EC-2B | Adenosquamous carcinoma, 2 | 30 | 8–39 | 0.09–0.44 | Rantanen *et al.* (1993*a*) |
| UT-EC-3 | Adenocarcinoma, 3 | 28 | 28–40 | 0.076–0.30 | Rantanen *et al.* (1993*b*) |

[a]Under characterisation (S. Grénman).

Carboplatin (Paraplatin) was dissolved in growth medium as a stock solution of 100 $\mu g\ ml^{-1}$ and sterilised by pressing through a 0.22 $\mu m$ filter. The final dilutions 0.05–2.5 $\mu g\ ml^{-1}$ were made immediately before use, and new stock solutions were made for each experiment.

### *Clonogenic assay*

The 96-well clonogenic assay based on limiting dilutions was used. The assay has been described earlier in detail (Grénman *et al.*, 1989; Rantanen *et al.*, 1993*b*). A minimum of three experiments including duplicate plates were performed for each cell line. The cells were harvested with trypsin–EDTA to obtain a single-cell suspension, counted and diluted in Ham's F-12 medium containing 15% fetal bovine serum (FBS) or newborn bovine serum (NBS). With a cell suspension containing 4,167 cells $ml^{-1}$ and diluted in 25 ml of growth medium, a concentration of two cells per well is achieved by applying 100 $\mu l$ to each well with an octapipette (Costar). The number of cells plated per well was adjusted according to the plating efficiency (PE) of the cell line. After plating into the 96-well plate the cells were allowed to attach for 24 h at 37°C in an incubator with a water vapour-saturated atmosphere containing 5% carbon dioxide. Twenty-four hours after plating, 100 $\mu l$ of growth medium containing the desired concentration of cisplatin or carboplatin was added to the wells.

To obtain dose–response curves for cisplatin and carboplatin, the drug solutions were allowed to remain in the plates during the whole incubation period. The plates were kept in the incubator for 4 weeks, after which time the number of wells containing coherent, living colonies (a colony consisting of 32 cells or more) was counted using an inverted phase-contrast microscope.

### *Data analysis*

Plating efficiency (PE) was calculated using the formula PE = – ln (number of negative wells/total number of wells)/ number of cells plated per well (Thilly *et al.*, 1980). The fraction survival data as a function of the cisplatin or carboplatin dose were fitted by linear quadratic equation. A microcomputer program was used to fit data $F = \exp[-(\alpha D + \beta D^2)]$. The comparison of drug sensitivity was made using $IC_{50}$ values (50% inhibition of surviving fraction), which were obtained from the fitted dose–response curves.

### Results

The PE values of the cell lines are listed in Table I. Dose–response curves for each cell line were obtained by fitting the data points to the linear quadratic equation (Figure 1a–h). The $IC_{50}$ values of the cell lines obtained from the cisplatin and carboplatin dose–response curves are given in Table II. $IC_{50}$ values for cisplatin varied between 0.022 $\mu g\ ml^{-1}$ and 0.56 $\mu g\ ml^{-1}$, and $IC_{50}$ values for carboplatin between 0.096 $\mu g\ ml^{-1}$ and 1.20 $\mu g\ ml^{-1}$. The mean $IC_{50}$ values for cisplatin and carboplatin were 0.23 $\mu g\ ml^{-1}$ and 0.50 $\mu g\ ml^{-1}$ respectively.

The $IC_{50}$ values for carboplatin were mostly higher than the corresponding values for cisplatin. The only exception was the UM-EC-2 cell line, which had an $IC_{50}$ value of 0.19 $\mu g\ ml^{-1}$ for cisplatin and 0.16 $\mu g\ ml^{-1}$ for carboplatin. The range of the ratios between carboplatin $IC_{50}$ and cisplatin $IC_{50}$, 1.5:1 to 4.1:1, was rather narrow, but no constant ratio between these values could be noticed. The sensitivity for both platinum analogues varied considerably between individual cell lines. The correlation between $IC_{50}$ values for cisplatin and carboplatin and the inherent radiosensitivity of the cell lines were estimated by Pearson correlation. No correlation could be demonstrated comparing the results from the current study with the radiosensitivity of the same cell lines tested earlier (Rantanen *et al.*, 1992, 1993*a*, *b*).

### Discussion

*In vitro* data on the chemosensitivity of endometrial cancer are scanty partly because of the lack of suitable cell lines. We have recently established and characterised numerous endometrial adenocarcinoma cell lines (Grénman *et al.*, 1988*b*, 1990) and used them for testing *in vitro* radiosensitivity of this tumour type (Rantanen *et al.*, 1992, 1993*a*, *b*). These studies have been performed using the 96-well-plate clonogenic assay, which has proven to be suitable for testing both radiosensitivity and chemosensitivity of adenocarcinoma as well as squamous cell carcinoma cell lines (Grénman *et al.*, 1988*a*, 1989, 1991; Pekkola-Heino *et al.*, 1989, 1991, 1992*a*, *b*; Rantanen *et al.*, 1992, 1993*a*, *b*). In this study we tested the chemosensitivity of eight endometrial cancer cell lines and demonstrated considerable variability between individual cell lines.

**Figure 1** Sensitivity of eight endometrial adenocarcinoma cell lines for cisplatin (○) and carboplatin (●). The figures show the fraction survival curves as a function of cisplatin and carboplatin dose. The results are given as the average of the actual data points and the bars represent 0.5 s.d. The data were fitted by a linear quadratic equation to produce the fraction survival curves. **a**, RL95-2; **b**, KLE; **c**, UM-EC-1; **d**, UM-EC-2; **e**, UM-EC-3; **f**, UT-EC-2A; **g**, UT-EC-2B; **h**, UT-EC-3.

The cytotoxic effects of cisplatin and carboplatin were compared in a variety of cell lines, including ovarian carcinoma cell lines (Hills *et al.*, 1989; Fanning *et al.*, 1990; Dittrich *et al.*, 1993) and stomach and lung cancer cell lines (Takahashi *et al.*, 1987). The results obtained from *in vitro* chemosensitivity testing vary considerably and because of different methods and exposure times they cannot be directly compared. Jones *et al.* (1987) determined the chemosensitivities of fresh human endometrial tumour samples with a soft-agar clonogenic assay. They used continuous drug exposure and achievable peak plasma levels (2.5 μg $ml^{-1}$) and one-tenth peak plasma levels (0.25 μg $ml^{-1}$). Twenty-seven of 30 endometrial adenocarcinoma samples demonstrated 70% or more reduction in colony formation with peak plasma level of cisplatin, and the corresponding number for one-tenth peak plasma level was 10 out of 21. Nguyen *et al.* (1991) evaluated the chemosensitivity of uterine cancer cell lines using ATP bioluminescence assay and 90 min exposure to cisplatin or carboplatin. $IC_{50}$ values for carboplatin were higher in three cases (× 1.1–2.1) and $IC_{50}$ values for cisplatin were higher in three cases (× 1.3–10.0).

Our results show great variation in the $IC_{50}$ values of

**Table II** Chemosensitivity of eight endometrial adenocarcinoma cell lines to cisplatin and carboplatin expressed as $IC_{50}$ values (concentration causing 50% inhibition of clonogenic survival)

| Cell line | *Cisplatin* $IC_{50} \pm$ *s.d.* ($\mu g\, ml^{-1}$) | *Carboplatin* $IC_{50} \pm$ *s.d.* ($\mu g\, ml^{-1}$) |
|---|---|---|
| RL95-2 | 0.43 ± 0.13 | 1.20 ± 0.07 |
| KLE | 0.022 ± 0.003 | 0.096 ± 0.009 |
| UM-EC-1 | 0.31 ± 0.10 | 0.46 ± 0.16 |
| UM-EC-2 | 0.19 ± 0.08 | 0.16 ± 0.03 |
| UM-EC-3 | 0.13 ± 0.05 | 0.27 ± 0.12 |
| UT-EC-2A | 0.15 ± 0.07 | 0.63 ± 0.15 |
| UT-EC-2B | 0.56 ± 0.06 | 1.06 ± 0.35 |
| UT-EC-3 | 0.034 ± 0.005 | 0.10 ± 0.03 |

endometrial adenocarcinoma cell lines for both cisplatin and carboplatin. The $IC_{50}$ for cisplatin varied from 0.022 $\mu g\, ml^{-1}$ to 0.56 $\mu g\, ml^{-1}$. There was a 25-fold difference between the most resistant and the most sensitive cell line. This finding is consistent with the results of Hills *et al.* (1989) obtained from testing ovarian cancer cell lines. The $IC_{50}$ values for carboplatin varied from 0.096 $\mu g\, ml^{-1}$ to 1.20 $\mu g\, ml^{-1}$. The difference between the most resistant and the most sensitive cell line was 12-fold. The capacity to cause cell death *in vitro* is lower for a given dose for carboplatin than for cisplatin. In this study the mean $IC_{50}$ values were 0.23 $\mu g\, ml^{-1}$ for cisplatin and 0.50 $\mu g\, ml^{-1}$ for carboplatin. The ratio of carboplatin $IC_{50}$ to cisplatin $IC_{50}$ varied in our material from 1.5:1 to 4.4:1. These data fit with the current clinical practice of using carboplatin and cisplatin in the ratio of 3–4:1. However, Terheggen *et al.* (1988, 1991) reported that on a molar basis 6–18 times more carboplatin than cisplatin is required to obtain the same level of DNA and platinum interaction products in cancer patients. Their *in vitro* studies indicated a direct correlation between cisplatin- and carboplatin-induced cell kill and DNA adduct production (Terheggen *et al.*, 1990). The predictive value of DNA adduct production is still unevaluated, and further clinical studies are needed to confirm the equivalent clinical doses with regard to efficacy of these two platinum analogues.

In our results the difference between UT-EC-2A and UT-EC-2B $IC_{50}$ values for cisplatin and carboplatin is of interest. The UT-EC-2A cell line was established from the primary tumour and the UT-EC-2B cell line was established from a supraclavicular metastasis after the donor had received six courses of chemotherapy including cisplatin. Cisplatin-induced growth inhibition was remarkable in UT-EC-2A cultures, whereas the UT-EC-2B cell line was the most resistant cell line to cisplatin. UT-EC-2B cells were also found to be highly resistant to carboplatin. These findings could be explained by selection of platinum-resistant cells in the donor's tumour during treatment with platinum-containing chemotherapy.

Results obtained by Kelland and Tonkin (1989) suggest a positive correlation between the chemo- and radiosensitivity of squamous cell carcinoma lines of the uterine cervix. Furthermore, a positive correlation between response to chemotherapy and subsequent response to radiotherapy has been reported in a group of patients with locally advanced cervical cancer (Kirsten *et al.*, 1987). We have previously tested the radiosensitivity of the endometrial cancer cell lines used in this study (Rantanen *et al.*, 1992, 1993*a*, *b*). Therefore, it was of interest to compare the chemosensitivity and radiosensitivity of individual cell lines. We could not find a correlation between cisplatin and carboplatin sensitivities and the intrinsic radiation sensitivity of the eight endometrial cancer cell lines.

Concomitant use of radiation and chemotherapy in the treatment of radioresistant tumours is one possibility to achieve better outcome in these patients. Owing to the widespread clinical use of platinum analogues in the treatment of advanced or recurrent endometrial cancer, it seems logical to evaluate their use also as radiosensitisers. Before planning schedules for chemoradiotherapy it is important to know the *in vitro* sensitivities of the drugs used for the tumour type in question. Without basic knowledge obtained from *in vitro* studies it is difficult to determine optimal doses and timing between radiation and the exposure to chemotherapeutic agent.

This study was supported by grants from the South-Western Division of the Finnish Cancer Society and the Turku University Foundation. We are grateful to Mrs Marita Potila for her invaluable and highly skilled assistance in performing the experiments. Cisplatin (Platinol) and carboplatin (Paraplatin) were generously provided by Orion Corporation Farmos, Turku, Finland.

### References

CHOI, K.N., ROTMAN, M., AZIZ, H., POTTERS, L., STARK, R. & ROSENTHAL, J.C. (1991). Locally advanced paranasal sinus and nasopharynx tumors treated with hyperfractionated radiation and concomitant infusion cisplatin. *Cancer*, **67**, 2748–2752.

DEWIT, L. (1987). Combined treatment of radiation and cis-diamminedichloroplatinum(II): a review of experimental and clinical data. *Int. J. Radiat. Oncol. Biol. Phys.*, **13**, 403–426.

DITTRICH, C., SEVELDA, P., BAUR, M., MARTH, C., HUDEC, M., VAVRA, N., GRUNT, T., FAZENY, B. & SALZER, H. (1993). *In vitro* and *in vivo* evaluations of the combination of cisplatin and its analogue carboplatin for platinum dose intensification in ovarian carcinoma. *Cancer*, **71**, 3082–3090.

DUNTON, C.J., PFEIFER, S.M., BRAITMAN, L.E., MORGAN, M.A., CARLSON, J.A. & MIKUTA, J.J. (1991). Treatment of advanced and recurrent endometrial cancer with cisplatin, doxorubicin, and cyclophosphamide. *Gynecol. Oncol.*, **41**, 113–116.

FANNING, J., BIDDLE, W.C., GOLDROSEN, M., CRICKARD, K., CRICKARD, U., PIVER, S. & FOON, K.A. (1990). Comparison of cisplatin and carboplatin cytotoxicity in human ovarian cancer cell lines using the MTT assay. *Gynecol. Oncol.*, **39**, 119–122.

GRÉNMAN, S.E., VAN DYKE, D.L., WORSHAM, M.J., DEL ROSARIO, F., ROBERTS, J.A., MCCLATCHEY, K.D., SCHWARTZ, D.L., BABU, R. & CAREY, T.E. (1988*a*). UM-EC-1, a new hypodiploid human cell line derived from a poorly differentiated endometrial cancer. *Cancer Res.*, **48**, 1864–1873.

GRÉNMAN, R., BURK, D., VIROLAINEN, E., WAGNER, J.G., LICHTER, A.S. & CAREY, T.E. (1988*b*). Radiosensitivity of head and neck cancer cells *in vitro*. *Arch. Otolaryngol. Head Neck. Surg.*, **114**, 427–431.

GRÉNMAN, R., BURK, D., VIROLAINEN, E., BUICK, R.N., CHURCH, J., SCHWARTZ, D.R. & CAREY, T.E. (1989). Clonogenic cell assay for anchorage-dependent squamous carcinoma cell lines using limiting dilution. *Int. J. Cancer*, **44**, 131–136.

GRÉNMAN, S.E., WORSHAM, M.J., VAN DYKE, D.L., ENGLAND, B., MCCLATCHEY, K.D., BABU, R., ROBETS, J.A., MÄENPÄÄ, J. & CAREY, T.E. (1990). Establishment and characterization of UM-EC-2, a tamoxifen-sensitive, estrogen receptor-negative human endometrial carcinoma cell line. *Gynecol. Oncol.*, **37**, 188–199.

GRÉNMAN, R., CAREY, T.E., MCCLATCHEY, K.D., WAGNER, J.G., PEKKOLA-HEINO, K., SCHWARTZ, M.S., WOLF, G.T., LACIVITA, L.P., HO, L., BAKER, S.R., KRAUSE, C.J. & LICHTER, A.S. (1991). *In vitro* radiation resistance among cell lines established from patients with squamous cell carcinoma of the head and neck. *Cancer*, **67**, 2741–2747.

HILLS, C.A., KELLAND, L.R., SIRACKY, G.A.J., WILSON, A.P. & HARRAP, K.R. (1989). Biological properties of ten human ovarian carcinoma cell lines: calibration *in vitro* against four platinum complexes. *Br. J. Cancer*, **59**, 527–534.

HOFFMAN, M.S., ROBERTS, W.S., CAVANAGH, D., PRAPHAT, H., SOLOMON, P. & LYMAN, G.H. (1989). Treatment of recurrent and metastatic endometrial cancer with cisplatin, doxorubicin, cyclophosphamide, and megestrol acetate. *Gynecol. Oncol.*, **35**, 75–77.

JACOBS, M.C., EISENBERGER, M., MIN CHU, O.H., SINIBALDI, V., GRAY, W., ELIAS, G. & SALAZAR, O.M. (1989). Carboplatin (CBDCA) and radiotherapy for stage IV carcinoma of the head and neck: a phase I–II study. *Int. J. Radiat. Oncol. Biol. Phys.*, **17**, 361–363.

JONES, C.M., WELANDER, C.E., BERENS, M.E. & HOMESLEY, H.D. (1987). *In vitro* growth characteristics and chemosensitivities of endometrial cancer using a soft agar clonogenic assay. *Obstet. Gynecol.*, **69**, 237-241.

KELLAND, L.R. & TONKIN, K.S. (1989). *In vitro* chemosensitivity of four new carcinoma of the cervix cell lines: relationship to radiosensitivity. *Eur. J. Cancer Clin. Oncol.*, **25**, 1211-1218.

KIRSTEN, F., ATKINSON, K.H., COPPLESON, J.V.M., ELLIOTT, P.M., GREEN, D., HOUGHTON, R., MURRAY, J.C., RUSSELL, P., SOLOMON, H.J., FRIEDLANDER, M., SWANSON, C.E. & TATTERSALL, M.H.H. (1987). Combination chemotherapy followed by surgery or radiotherapy in patients with locally advanced cervical cancer. *Br. J. Obstet. Gynecol.*, **94**, 583-588.

LITTERS, C.L., GRAM, T.E., DEDRICK, R.L., LEROY, A.F. & GUARINO, A.M. (1976). Distribution and disposition of platinum following intravenous administration of *cis*-diamminedichloroplatinum(II) (NSC 119875) to dogs. *Cancer Res.*, **36**, 2340-2344.

LITTERS, C.L., TORRES, I.J. & GUARINO, A.M. (1977). Plasma levels and organ distribution of platinum in the rat, dog, and dogfish shark following single intravenous administration of *cis*-dichlorodiammineplatinum(II). *J. Clin. Hematol. Oncol.*, **7**, 169-179.

NGUYEN, H.A., SEVIN, B.-U., AVERETTE, H.E., PERRAS, J., DONATO, D. & PENALVER, M. (1991). Comparative evaluation of single and combination chemotherapy in uterine cancer cell lines. *Gynecol. Oncol.*, **42**, 227-232.

NGUYEN, H.N., SEVIN, B.-U., AVERETTE, H.E., GOTTLIEB, C., PERRAS, J., DONATO, D. & PENALVER, M. (1993). Radiosensitization of uterine cancer cell lines by cytotoxic agents. *Gynecol. Oncol.*, **48**, 16-22.

PEKKOLA-HEINO, K., KULMALA, J., GRÉNMAN, S., CAREY, T.E. & GRÉNMAN, R. (1989). Radiation response of vulvar squamous cell carcinoma (UM-SCV-1A, UM-SCV-1B, UM-SCV-2, and A-431) cells *in vitro*. *Cancer Res.*, **49**, 4876-4878.

PEKKOLA-HEINO, K., KULMALA, J., KLEMI, P., LAKKALA, T., AITASALO, K., JOENSUU, H. & GRÉNMAN, R. (1991). Effects of radiation fractionation on four squamous cell carcinoma lines with dissimilar inherent radiation sensitivity. *J. Cancer Res. Clin. Oncol.*, **117**, 597-602.

PEKKOLA-HEINO, K., KULMALA, J. & GRÉNMAN, R. (1992*a*). Carboplatin-radiation interaction in squamous carcinoma cell lines. *Arch. Otolaryngol. Head Neck Surg.*, **118**, 1312-1315.

PEKKOLA-HEINO, K., KULMALA, J. & GRÉNMAN, R. (1992*b*). Sublethal damage repair in squamous cell carcinoma cell lines. *Head & Neck*, **14**, 196-199.

RANTANEN, V., GRÉNMAN, S., KULMALA, J., SALMI, T. & GRÉNMAN, R. (1992). Radiation sensitivity of endometrial carcinoma *in vitro*. *Gynecol. Oncol.*, **44**, 217-222.

RANTANEN, V., GRÉNMAN, S., KULMALA, J., LAKKALA, T., SAJANTILA, A., KLEMI, P. & GRÉNMAN, R. (1993*a*). Characterization and radiosensitivity of UT-EC-2A and UT-EC-2B, two new highly radiosensitive endometrial cancer cell lines derived from a primary and metastatic tumor of the same patient. (submitted).

RANTANEN, V., GRÉNMAN, S., KULMALA, J., ALANEN, K., LAKKALA, T. & GRÉNMAN, R. (1993*b*). Sublethal damage repair after fractionated irradiation in endometrial cancer cell lines tested with the 96-well plate clonogenic assay (submitted).

RICHARDSON, G.S., DICKERSIN, G.R., ATKINS, L., MACLAUGHLIN, D.T., RAAM, S., MERK, L.P. & BRADLEY, F.M. (1984). KLE: A cell line with estrogen receptor derived from undifferentiated endometrial cancer. *Gynecol. Oncol.*, **17**, 213-230.

TAKAHASHI, H., SASAKI, Y., SAIJO, N., SAKURAI, M., NAKANO, H., NAKAGAWA, K., HOSHI, A., JETT, J.R. & HONG, W.-S. (1987). *In vitro* colony inhibition of carboplatin against stomach and lung cancer cell lines in comparison with cisplatin. *Cancer Chemother. Pharmacol.*, **19**, 197-200.

TERHEGGEN, P.M.A.B., DIJKMAN, R., BEGG, A.C., DUBBELMAN, R., FLOOT, B.G.J., HART, A.A.M., HART, A.M. & DEN ENGELSE, L. (1988). Monitoring of interaction products of *cis*-diamminedichloroplatinum(II) and *cis*-diammine(1,1-cyclobutanedicarboxylato)platinum(II) with DNA in cells from platinum-treated cancer patients. *Cancer Res.*, **48**, 5597-5603.

TERHEGGEN, P.M.A.B., EMONDT, J.Y., FLOOT, B.G.J., DIJKMAN, R., SCHRIER, P.I., DEN ENGELSE, L. & BEGG, A.C. (1990). Correlation between cell killing by *cis*-diamminedichloroplatinum(II) in six mammalian cell lines and binding of a *cis*-diamminechloroplatinum(II)-DNA antiserum. *Cancer Res.*, **50**, 3556-3561.

TERHEGGEN, P.M.A.B., BEGG, A.C., EMONDT, J.Y., DUBBELMAN, R., FLOOT, B.G.J. & DEN ENGELSE, L. (1991). Formation of interaction products of carboplatin with DNA *in vitro* and in cancer patients. *Br. J. Cancer*, **63**, 195-200.

THIGPEN, T., VANCE, R., LAMBUTH, B., BALDUCCI, L., KHANSUR, T., BLESSING, J. & MCGEHEE, R. (1987). Chemotherapy for advanced or recurrent gynecologic cancer. *Cancer*, **60**, 2104-2116.

THILLY, W.G., DELUCA, J.G., FURTH, E.E., HOPPE, H., KADEN, D.A., KROLENSKI, J.J., LIBER, H.L., SKOPEK, T.R., SLAPIKOFF, S.A., TIZARD, R.J. & PENMAN, B.W. (1980). Gene-locus mutation assay in diploid human lymphoblast lines. In *Chemical Mutagens*, Vol. 6, De Serpes, F.J. & Hollaender, A. (eds), pp. 331-363. Plenum: New York.

WAY, D.I., GROSSO, D.S., DAVIS, J.R., SURWIT, E.A. & CHRISTIAN, C.D. (1983). Characterization of a new human endometrial carcinoma (RL95-2) established in tissue culture. *In Vitro*, **19**, 147-158.